# Modulating the Conductivity of Light-Responsive Ionic Liquid Crystals

**DOI:** 10.3390/molecules29184459

**Published:** 2024-09-20

**Authors:** Umama Bendaoud, Pradip K. Bhowmik, Si L. Chen, Haesook Han, Seonghyeok L. Cox, Jasmin Liebsch, M. Blanca Ros, Thamil Selvi Velayutham, Nurul Fadhilah Kamalul Aripin, Alfonso Martinez-Felipe

**Affiliations:** 1 Chemical Processes and Materials Research Group, Just Transition Lab, Centre for Energy Transition, School of Engineering, University of Aberdeen, King’s College, Aberdeen AB24 3UE, UK; umamabendaoud98@gmail.com (U.B.); j.liebsch.20@abdn.ac.uk (J.L.); 2 Department of Chemistry and Biochemistry, University of Nevada Las Vegas, 4505 S. Maryland Parkway, Box 454003, Las Vegas, NV 89154, USA; pradip.bhowmik@unlv.edu (P.K.B.); chens19@unlv.nevada.edu (S.L.C.); hanh3@unlv.nevada.edu (H.H.); coxs4@unlv.nevada.edu (S.L.C.); 3 Department of Chemistry, University of Aberdeen, King’s College, Aberdeen AB24 3UE, UK; 4 Instituto de Nanociencia y Materiales de Aragón, Departamento de Química Orgánica, Facultad de Ciencias, Universidad de Zaragoza-CSIC, Campus San Francisco, E-50009 Zaragoza, Spain; bros@unizar.es; 5 Low Dimensional Materials Research Center, Department of Physics, Faculty of Science, Universiti Malaya, Kuala Lumpur 50603, Malaysia; t_selvi@um.edu.my; 6 School of Chemical Engineering, College of Engineering, Universiti Teknologi MARA, Shah Alam 40450, Malaysia; fadhilah9413@uitm.edu.my; 7 Department of Chemistry, School of Natural and Computing Sciences, University of Aberdeen, King’s College, Aberdeen AB24 3UE, UK

**Keywords:** ionic liquid crystals, azobenzenes, photoisomerisation, ionic conductivity, energy conversion and storage

## Abstract

In this work, we describe the phase behaviour and the dielectric and conductivity response of new light-responsive ionic liquid crystals, ILCs, which can be applied as controllable electrolytes. The materials include two different dicationic viologens, the asymmetric 6BP18 and the symmetric EV2ON(Tf)_2_, containing bistriflimide as the counterions, mixed with 5% and 50% molar, respectively, of one new photoresponsive mesogen called CNAzO14. These mixtures exhibit liquid crystal behaviour, light responsiveness through the *E*-*Z* photoisomerisation of the azobenzene groups in CNAzO14, and strong dielectric responses. The 5%-CNAzO14/Ev2ON(Tf)_2_ mixture displays direct current conductivities in the 10^−7^ S·cm^−1^ range, which can be increased by a two-fold factor upon the irradiation of UV light at 365 nm. Our findings set the grounds for designing new smart ionic soft materials with nanostructures that can be tuned and used for energy conversion and storage applications.

## 1. Introduction

Ionic liquids (ILs) are versatile materials that contain organic cations and inorganic/organic anions and have melting transitions (*T*_m_) typically lower than 100 °C, and on some occasions well below ambient temperatures. Their combination of physical properties (low vapour pressure, non-flammability, non-volatility, high thermal stability, and conductivity) and molecular design flexibility (through organic functionalisation) makes them suitable candidates for a variety of applications. ILs have been postulated as green solvents (for synthesis and extraction processes), additives (inhibitors, surface-active agents, and plasticisers), and components in advanced materials [1,2,3,4,5].

In particular, due to their low glass transition temperatures (*T*_g_) in the −100 to −50 °C range, ILs display a high free volume at ambient temperatures that promotes molecular (and potentially ionic) mobility, making them suitable as electrolytes in energy conversion and storage applications [6,7,8,9]. In the past years, our group has investigated new multi-charged IL electrolytes, which combine low viscosity and high ionic conductivity, including dicationic stilbazolium salts [10], asymmetric viologens [11], and symmetric extended viologens [12]. Under certain conditions, ILs can form liquid crystalline phases (mesophases), in which they can flow whilst still retaining a certain degree of molecular positional or orientational order, resulting in ionic liquid crystals (ILCs) [4,5,13]. The formation of smectic [14], columnar [15], or bicontinuous [16] phases in liquid crystals can facilitate ionic transport through anisotropic domains with high molecular mobility [17,18,19,20,21,22,23,24,25]. The presence of ionic moieties in ILCs can further enhance conductivity through their nanostructures, and the development of highly conducting ILCs can be considered as a nascent topic with massive opportunities in technology applications. In particular, the presence of conjugated bi- or multi-pyridyl groups makes viologen-based materials excellent candidates to form liquid crystal phases, due to the ionic and localised conjugation that promotes “soft” intermolecular interactions [26,27,28,29,30]. However, the strong molecular interactions between ionic and polarisable groups can inhibit molecular motions and limit the performance of ILCs as electrolytes.

**Figure 1:**
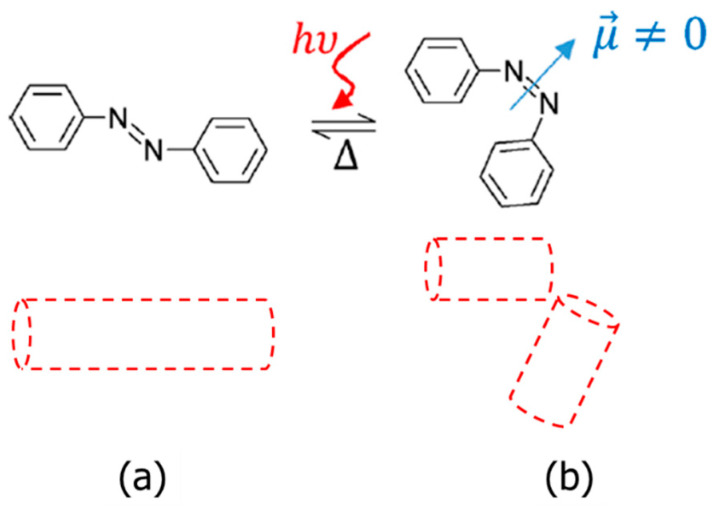
Chemical structure of the azobenzene group: (**a**) *E*-isomer (*trans*, ground state) and (**b**) *Z*-isomer (*cis*, excited state), showing the *E*-*Z* photoisomerisation (by UV light irradiation), the thermally activated *Z*-*E* relaxation (∆), and a representation of the dipole moment in the *Z*-isomer, $\vec{\mu }$.

In this work, we will apply light as an external stimulus to trigger and promote charge mobility in new light-responsive ILC materials, through the *E*(*trans*)-to-*Z*(*cis*) photoisomerisation of azobenzene groups. In its ground state, the linear *E*-isomer is prevalent, Figure 1a, and is normally compatible with the ordered mesophases shown by LCs. Upon the irradiation of UV light (normally in the ~365 nm range), the resulting excited *Z*-isomer adopts some molecular curvature that disrupts the local order, even leading to isothermal phase transitions, as shown in Figure 1b [31,32]. Some recent work has proved that the steric changes caused by *E*-*Z* photoisomerisation can be useful in inducing the reversible modulation of conductivity in polyelectrolytes [33,34] and ionic liquids [35]. However, whilst photoinduced effects on liquid crystalline materials have been broadly studied over the past years [36], their application to ionic liquid crystals remains mainly unexplored, despite the potential that photoresponsive ILCs have as advanced materials [37,38,39,40]. We envisage that the present methodology and results will open new fields for ILC electrolytes with light-responsive properties.

## 2. Experimental Procedure

### 2.1. Materials

**Figure 2:**
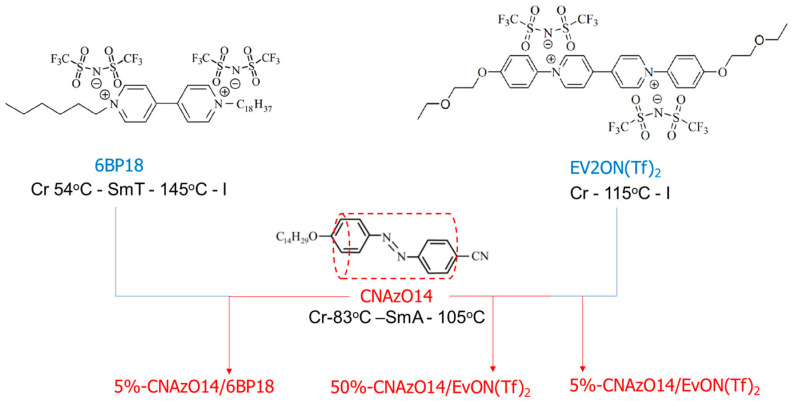
Chemical structures and simplified thermal ranges (values on cooling) of the three pristine compounds used to prepare the light-responsive mixtures under study.

We have selected two ILs containing bistriflimide as the counterions, namely 6BP18 and EV2ON(Tf)_2_, based on their promising ionic conductivities [10,12]; see Figure 2. In order to yield photoresponsive materials, we have doped these ILs with 5% (molar%) of a new rod-like azobenzene derivative, CNAzO14, with the aim to use the minimum amount of dopant to yield light responsiveness [41]. The synthesis and full characterisation of the pristine materials are summarised in the Supplementary Information. 6BP18 forms a smectic T phase, SmT, in a broad range of temperatures (from 54 °C to 145 °C (see Figure S2 and also [11]), and Ev2ON(Tf)_2_ does not show mesophormic behaviour and does not crystallise from the melt on cooling at a rate of 10 °C·min^−1^. CNAzO14 forms a smectic A phase (from 83 °C to 105 °C; see Figure S3) in comparable ranges to those observed for its C16 analogous [42]. The formation of smectic phases by CNAzO14 and 6BP18 is explained by the microphase separation between the polar rigid cores of these molecules and their alkyl (non-polar) chains [11,43].

The mixtures were prepared by weighting the corresponding amounts of the compounds and adding a few mL of a common solvent (tetrahydrofuran, THF) inside a vial and stirring at room temperature for 24 h. The solvent in the resulting solutions was then allowed to evaporate, and the powder was further dried in a vacuum oven for several days.

### 2.2. Techniques and Methods

The pristine materials and the mixtures were filled from their melt into commercial ITO-coated (Indium Tin Oxide) glass cells (SG100A080uG180, Instec, see Figure S4) by capillary action. The cells have an active area of $A=100\;mm^{2}$, a thickness of t=8 μm, and a resistance of 100 Ω. The parallel capacitance, $C_{p}$, is given by

(1) $$C_{p}=\frac{\varepsilon^{\prime }\varepsilon_{O}A}{t}$$

where $\varepsilon_{o}=8.854\times 10^{-12}\;F\cdot m^{-1}$ is the permittivity of the vacuum, and $\varepsilon^{\prime }$ is the dielectric permittivity of the sample. The ITO cells were then connected to the frequency response analyser (FRA) using two aluminium foils attached to the sides of the glass cells with conductive silver paint.

A Linkam THMS 600 heating stage, controlled by a TMS 91 unit, maintained the sample temperature with ±0.1 °C accuracy. Phase behaviour observations were conducted using an Olympus BX53M polarised optical microscope (POM) equipped with a cross-polariser configuration. Complex impedance spectroscopy measurements were performed using a Solartron Modulab XM frequency response analyzer (FRA). Frequency sweeps from 0.01 to 10^6^ Hz were conducted at various isothermal steps during cooling from the melt, applying an alternating voltage of 1 V_rms_. Additional time-domain measurements were performed at a fixed frequency of 1 Hz to complement the frequency sweep data. The complex dielectric permittivity is shown in Equation (2),

(2) $$\varepsilon^{*}\left(\omega \right)=\varepsilon^{\prime }-j\varepsilon^{\prime \prime }$$

where ε′ and ε″ are the real and imaginary dielectric permittivity, respectively. The complex conductivity was obtained from

(3) $$\sigma^{*}\left(\omega \right)=\sigma^{\prime }-j\sigma^{\prime \prime }$$

where

(4) $$\sigma^{\prime }=\omega \epsilon_{O}\varepsilon^{\prime \prime },$$

and

(5) $$\sigma^{\prime \prime }=\omega \epsilon_{O}\varepsilon^{\prime },$$

with ω = 2 πf as the angular frequency (rad·s^−1^).

UV irradiation was performed using a Dymax Bluewave QX4 LED system equipped with a Dymax ACCU-CAL 50-LED intensity controller. Samples were exposed to 365 nm light at a calibrated intensity of 260 mW·cm^−2^. UV–visible spectra were subsequently recorded using an Agilent Cary 50 Spectrophotometer.

## 3. Results and Discussion

### 3.1. Phase Behaviour and Conductivity

**Figure 3:**
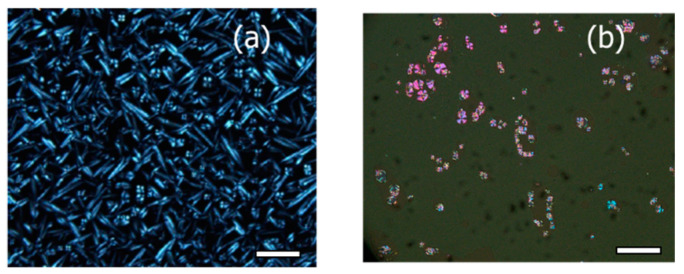
Polarised optical micrographs (POMs) corresponding to (**a**) 5%-CNAzO14/6BP18, at 130 °C, and (**b**) 50%-CNAzO14/EV2ON(Tf)_2_, at 105 °C. White bars correspond to 20 µm.

The 5%-CNAzO14/6BP18 mixture exhibits a smectic phase between 127 °C and 119 °C upon cooling from the isotropic state, as evidenced by the formation of conical fan textures observed under the POM; see Figure 3a. Given the similarity with the POM texture obtained for pristine 6BP18, Figure S2, we suggest that 5%-CNAzO14/6BP18 forms smectic T phases [11]. On the other hand, the analogous 5%-CNAzO14/Ev2ON(Tf)_2_ mixture does not exhibit liquid crystalline phases, which is explained by the rigidity of the extended four-ring core and the relatively short ethyloxy chains (*n* = 2). To overcome this fact, we increased the concentration of the mesogenic CNAzO14, up to 50% (molar%). The 50%-CNAzO14/EV2ON(Tf)_2_ mixture exhibits birefringent droplets upon cooling from the isotropic state, indicative of a liquid crystalline phase between 130 °C and 100 °C; see Figure 3b. The images resemble focal-conic fan textures typical of smectic phases, which would be compatible with the SmA exhibited by CNAzO14, even though further experiments would be necessary to confirm the mesophase unambiguously. From a structural point of view, the introduction of the C14 alkyl chains has a plasticising effect and somehow counteracts the rigidity of the four-ring core of the EV2ON(Tf)_2_ molecules.

All the materials exhibit a strong dielectric response, based on the presence of highly polarisable groups; see Figure S5. The dielectric spectra of the samples, illustrated by their loss factor relative to the vacuum permittivity value, ε_r_″, are dominated by a main peak through the smectic range, which shifts to lower frequencies upon cooling down, and splits on crystallisation. This process has been related to the rotation of the main molecular core, analogous to the so-called β-relaxation in rod-like liquid crystals [11,44,45,46]. Hence, the polarisation of the materials is linked to their molecular mobility, which will ultimately depend on their phase behaviour.

**Figure 4:**
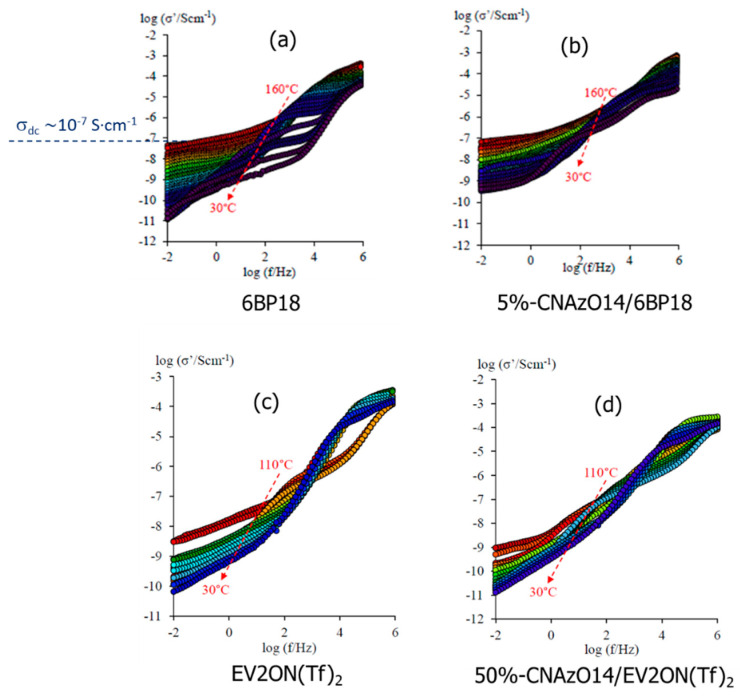
Isothermal Bode plots of the real conductivity, σ′, of (**a**) 6BP18; (**b**) 5%-CNAzO14/6BP18; (**c**) Ev2ON(Tf)_2_; and (**d**) 50%-CNAzO14/Ev2ON(Tf)_2_, measured on cooling from their isotropic melts to their crystal phases.

The conductivity of the ILCs was calculated using Equations (3)–(5), and the results for the real component of the complex conductivity, σ′, are shown in Figure 4. Both 5%-CNAzO14/6BP18 and the pristine IL 6BP18 display direct current conductivity, σ_dc_, evidenced by the formation of plateaus in the double logarithmic plots, visible in Figure 4a,b, respectively. Both materials reach a maximum of σ_dc_~10^−7^ S·cm^−1^, estimated by the extrapolation of σ′ to f → 0, and, interestingly, the inclusion of the azobenzene units of CNAzO14 seems to enhance the conductivity of 6BP18 at low temperatures. The EV2ON(Tf)_2_ materials, on the other hand, display a lower conductivity than their 6BP18 analogue, with ill-defined plateaus in the σ_dc_ ~ 10^−9^ S·cm^−1^ range. This difference can be justified, at least in part, by the shorter aromatic core to aliphatic chains ratio of 6BP18 with respect to EV2ON(Tf)_2_. Whilst the inclusion of CNAzO14, with a longer aliphatic/aromatic ratio, is enough to promote liquid crystallinity, recall Figure 3b, it seems that it cannot promote sufficient ionic mobility. It is worth noting, moreover, that the 50%-CNAzO14/EV2ON(Tf)_2_ sample only contains 50% of ionic molecules.

### 3.2. Light-Responsive Properties

**Figure 5:**
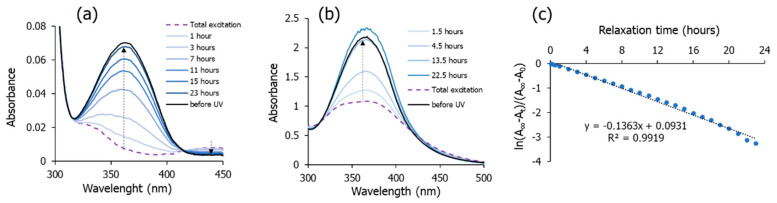
UV-vis spectra of the ILC mixtures measured for ~10^−5^ M THF solutions at room temperature, before and at different times after light irradiation (260 mW·cm^−2^; 365 nm) for (**a**) 5%-CNAzO14/6B18 and (**b**) 50%-CNAzO14/Ev2ON(Tf)_2_; arrows indicate signal recovery (relaxation) while samples were kept in the dark. (**c**) Relaxation kinetics of 5%-CNAzO14/6B18: a plot of the maximum absorbance values (~360 nm) of the UV–visible spectra at different times while kept in the dark (A_t_), after light irradiation (A_0_), and until the curves recover their initial values prior to irradiation (A_∞_). Estimation of the half-life time, t_1/2_ = 5 h and 5 min. No noticeable changes were observed at higher wavelengths.

As it was intended, the introduction of CNAzO14 promotes photoresponsive character in the mixtures, due to *E*-*Z* photoisomerisation of its azobenzene groups. The excitation (*E*-*Z*) and relaxation (*Z*-*E*) processes were confirmed by monitoring the UV–visible spectra of the THF solutions of the samples (~10^−5^ M), before and after irradiation at 365 nm, at room temperature. Figure 5a,b summarise the results corresponding to 5%-CNAzO14/6B18 and 50%-CNAzO14/Ev2ON(Tf)_2_, respectively. All solutions show a strong band associated with the lowest-energy π* ← *π* transition in the *E*-isomer (~365 nm) and a much smaller intensity absorption band in the visible region (~440 nm), assigned to a weak π* ← *n* transition in the *Z*-isomer [31]. After irradiation with UV light (260 mW·cm^−2^), azobenzene units undergo *E*-*Z* photoisomerisation, evidenced by the rapid decrease in the 365 nm band, accompanied by a slight increase in the 440 nm region. It is worth noting that, whilst the π* ← *π* transition of 5%-CNAzO14/6BP18 is fully suppressed by irradiation, Figure 5a, the band corresponding to 50%-CNAzO14/Ev2ON(Tf)_2_ preserves some “residual” value, and we will return to this observation later.

After irradiation ceases, samples undergo thermally activated *Z*-*E* back relaxation while kept in the dark, and the original UV-vis signals are recovered after 24 h. The kinetics of relaxation can be studied by monitoring the maximum of the ~365 nm peak, and this was illustrated for 5%-CNAzO14/6BP18 in Figure 5c. Whilst excitation occurs almost immediately, relaxation requires hours, due to the low temperature involved in such a thermally activated process. The linear recovery in the logarithmic scale is typical of first-order processes and is consistent with precedents in the literature, including other liquid crystalline materials [47,48]. The much slower relaxation process, compared to excitation, can be explained by strong viscosity forces that may be present even in the solution.

**Figure 6:**
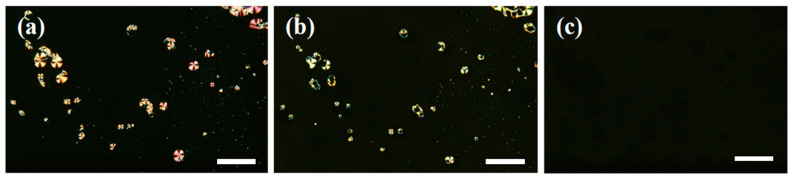
Polarised optical micrographs (POMs) obtained for 50%-CNAzO14/Ev2ON(Tf)_2_ before (**a**), during, (**b**) and while maintaining (**c**) UV irradiation at 365 nm (260 mW·cm^−2^, 105 °C). White bars correspond to 20 μm.

*E*-*Z* photoisomerisation can also take place in bulk, which is more relevant for device applications. When we exposed the ITO cell of 50%-CNAzO14/Ev2ON(Tf)_2_ at its mesophase range (T > 100 °C) to UV light (365 nm, 260 mW·cm^−2^), we observed the disappearance of birefringent regions when looked at under the polarised optical microscope, as seen in the sequence in Figure 6. This denotes isothermal isotropisation, i.e., the mixture undergoes a phase transition to an isotropic melt from the mesophase, caused by the reduction in shape anisotropy in the presence of the less linear *Z*-isomers [49]. Oppositely, it was not possible to induce such an isothermal phase transition to 5%-CNAzO14/6BP18, even at temperatures sufficiently close to the clearing point, and the needle-type textures typical of the smectic T phase remained under similar UV exposure conditions; see Figure S6. These results can be explained, at least in part, by the higher concentration of photoresponsive CNAzO14 molecules in the 50%-CNAzO14/Ev2ON(Tf)_2_ mixture. However, if we revisit Figure 5b, we must consider that the light intensity (power) may not be enough to switch all the azobenzene groups in 50%-CNAzO14/Ev2ON(Tf)_2_. It is possible that only a small fraction of *Z*-isomers is necessary to destabilise the already narrow LC phase observed for this mixture, but also that photoisomerisation is more effective in bulk than in solution, even though it is considered a locally activated phenomenon [31].

**Figure 7:**
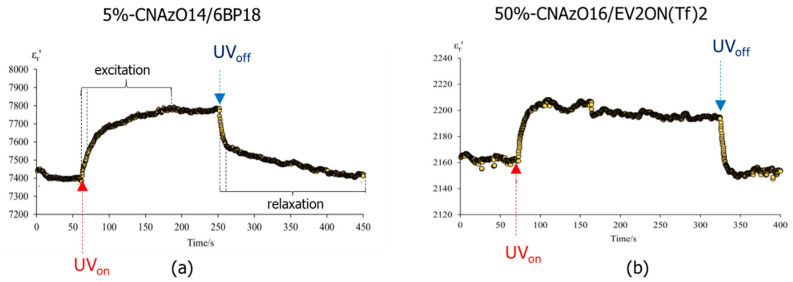
Dynamic response of the dielectric constant (relative to the vacuum value, ε′_r_ = ε′/ε_0_) to light irradiation with a UV lamp (intensity of 260 mW cm^−2^) corresponding to (**a**) 5%-CNAzO14/6BP18 and (**b**) 50%-CNAzO14/Ev2ON(Tf)_2_, measured in their crystal phases (30 °C). Vertical dotted lines delimitate the two-step process of excitation and relaxation.

**Figure 8:**
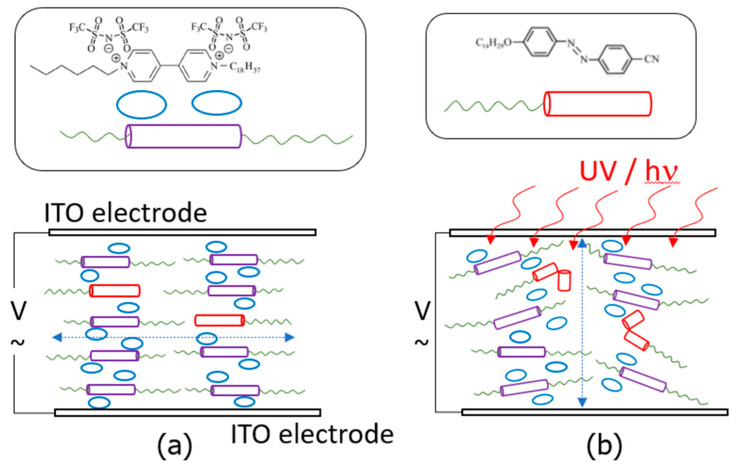
Molecular sketches representing the potential alignment of the ILCs in the ITO cells, taking 5%-CNAzO14/6B18 as a model: (**a**) planar alignment (no UV irradiation: all azobenzene groups are *E*-isomers); and (**b**) quasi-planar alignment, illustrating the partial disruption of the smectic order (under UV irradiation: some azobenzene groups photoisomerise to *Z*-isomers). Dotted lines indicate potential ionic channels.

Light irradiation also promotes variations in the dielectric response in the mixtures. Figure 7a,b display the dielectric constant (relative to the vacuum value, hereinafter ε′_r_ = ε′/ε_0_) of the two mixtures in the time domain, before and after irradiation at 365 nm near room temperature (30 °C). The dielectric constant of both samples undergoes an exponential-type increase (on illumination), before stabilising to a plateau value, followed by a decrease (after switching off), until relaxing back to their respective baseline values in equilibrium. Such a response is consistent with previous opto-dielectric effects reported in LC hosts with azobenzene guests [50,51]. Our results in Figure 7 can be explained in terms of molecular alignment and dipole moments. In the absence of external stimuli, the rigid cores of the three compounds in this study promote planar alignments of the molecules within the ITO cells, and the highly polarisable groups (e.g., cyanobiphenyl groups and ethoxy chains) may lie on average perpendicularly to the alternating electric field; see Figure 8a [52]. As a result, the dielectric response of such polar groups is somehow hindered. Under the application of light and following photoisomerisation, the disruption of the liquid crystalline order may also alter the molecular alignment in the ITO cells, and the reorientation of some polarisable groups results in an increase in the dielectric signal; see Figure 8b. This effect is (somehow) analogous to the application of strong fields in materials with positive anisotropy, studied for LCs [52]. The stronger dielectric response (ε′_r_) of the mixtures during irradiation may be also explained, at least in part, by contributions from dipole moments of the *Z*-isomers, which are larger than the near-to-zero moment of the linear *E*-isomer; recall Figure 1 [53,54]. However, this argument is only valid if the molecules are retaining certain planar orientations within the cells, preventing dipole cancellations. Even though we note that viologen-based materials can indeed show a photochromic response, normally used for luminescence applications (see for example, [55,56]), the changes in the π* ← *π* and π* ← *n* transitions reported above, and the absence of other transitions in the UV–visible spectra in our mixtures, strongly suggest that the dielectric response must be associated with the *E*-*Z* photoisomerisation of the azobenzene groups.

We note that the modulation of the mixtures’ dielectric constant observed in Figure 7 is quite fast, occurring in the seconds scale, even though the measurements were taken at their respective crystal phases. The 50%-CNAzO14/Ev2ON(Tf)_2_ mixture seems to undergo faster excitation and relaxation processes but with smaller relative variations in its ε′_r_ values. This somehow mirrors our findings in Figure 5b, which suggested that not all the azobenzene groups in CNAzO14 could undergo *E*-*Z* photoisomerisation under the current irradiation conditions. These results, however, must be taken carefully, since photoisomerisation can be very different in solutions and in bulk and depends on multiple factors (chromophore concentration, temperature, light irradiation, and time) [41].

Assuming that the planar order is kept in their crystal structures after cooling from their respective liquid crystalline phases, we speculate that the two-step process distinguishable for 5%-CNAzO14/6BP18 in Figure 7a could be associated with the two dielectric contributions explained above. A first rapid increase in ε′_r_ upon irradiation can be related to a fast *E*-*Z* photoisomerisation of the azobenzenes present in the mixture, followed by an asymptotic growth, which can be related to the reorientation of the molecules away from the planar alignment. The involvement of viscous effects in such motions can explain the longer times required to reach a steady state with constant ε′_r_ values, where a mixed population of *E* and *Z* isomers coexists in equilibrium under UV illumination, with dipole moments having different relative directions with respect to the ITO electrodes and their respective long molecular axes. Fairly symmetric relaxation processes occur upon switching off the UV light in Figure 7. Despite taking place at low temperatures, relaxation seems very rapid, and faster than that measured under solution in Figure 5a, potentially supported by cooperative forces that contribute to recovering the initial planar alignment.

**Figure 9:**
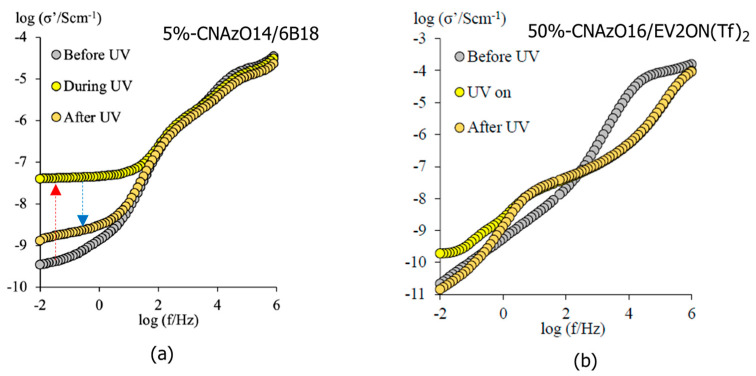
Isothermal Bode plots of the real conductivity, σ′, of (**a**) 5%-CNAzO14/6BP18 (30 °C) and (**b**) 50%-CNAzO14/Ev2ON(Tf)_2_ (40 °C), measured before, during, and after light irradiation (365 nm, 260 mW·cm^−2^). Dotted arrows indicate excitation (red) and relaxation (blue) processes. Measurements were taken after two minutes of irradiation start and stop, to allow the samples to reach a stationary state.

As expected, light irradiation also increases the conductivity of 5%-CNAzO14/6BP18, which undergoes a two-fold jump, prior to relaxing back to similar initial values after the light is switched off; see Figure 9a. We acknowledge that such an increase is remarkable, considering that it was measured at low temperatures in the crystal phase, when the molecular mobility is expected to be limited. In the past, we reported analogous phenomena in LC polymers [24] and bent-core LCs [48], and we attributed the increase in conductivity to the induction of local defects in the smectic structures. Such local “softening” would be comparable to partially breaking crosslinking in smectic networks, described by other authors [57]. We also speculated that continuous *E*-*Z*-*E* photoisomerisations leading to iso-mesophase micro-transitions could further improve the dielectric (and conductivity) signal [48]. Taking into account our models in Figure 8, the present planar alignment may not favour long-range conductivity between the electrodes, which could also explain our moderate σ_dc_ values compared to other liquid crystalline materials [58,59,60,61]. Alignment of smectic channels perpendicular to electrolytes will be paramount in yielding competitive conductivity values, even though this can be challenging in viscous phases formed by ionic liquids (this work), by block copolymers [23] or ionic LCs [11]. The more moderate conductivity increase in 50%-CNAzO14/Ev2ON(Tf)_2_ under similar experimental conditions, see Figure 9b, mirrors the dynamic response of the dielectric constant, ε′_r_, measured in Figure 7b, and the limiting effect of azobenzene concentration on light-induced conductivity.

## 4. Conclusions

The two ionic materials under study have shown LC behaviour and light responsiveness, through reversible *E*-*Z*-*E* photoisomerisation of the azobenzene group present in the CNAzO14 mesogen. The 50%-CNAzO14/Ev2ON(Tf)_2_ mixture exhibits LC (probably a smectic-type phase) in a narrow temperature range, which can be suppressed by UV light irradiation through a decrease in the anisotropy, indicating that the stability of the mesophase is not very high. The induction of its LC behaviour is based on the large concentration of CNAzO14 mesogens, but the light response is limited, probably due to the amount of energy required to photoisomerise their azobenzene groups. The 5%-CNAzO14/6BP18 mixture, on the other hand, shows a more promising control of the dielectric properties through light irradiation. Pristine 6BP18 already exhibited LC behaviour, and its smectic T phases are still maintained after the introduction of 5% molar of CNAzO14. Under our experimental conditions (365 nm, 260 mW·cm^−2^), UV light irradiation enhances the conductivity response of 5%-CNAzO14/6BP18 by two orders of magnitude. The observed phase transition might be attributed to a combination of factors including increased molecular dipole moments of *Z*-isomers, formation of locally disordered regions with enhanced molecular mobility, or disruption of molecular alignment due to micro-isotropic domains. The nature of these phenomena will be further investigated by designing in situ infrared spectroscopy and diffractometry experiments during light irradiation.

Even though our conductivity results are still far from those obtained with commercial electrolytes used in, for example, fuel cells or batteries (in the 10^−1^ S·cm^−1^ range), this work demonstrates that light illumination can be an excellent strategy to exert spatial control over the conductivity of new nanostructured electrolytes based on ionic LC, by using photoresponsive dopants. In fact, the dynamic (and reversible) behaviour observed at the crystal phases of the mixtures in Figure 7 and Figure 9 demonstrates the potential of these materials to be used in near-room-temperature conditions. Using ILs with higher conductivities, tuning the alignment of nanochannels, or promoting local mobility in ordered smectic layers can lead to new breakthroughs for functional and advanced ionic liquid crystalline materials for energy conversion and storage applications.

## Data Availability

Data are available upon request.

## Supplementary Materials

The following supporting information can be downloaded at https://www.mdpi.com/article/10.3390/molecules29184459/s1, 1. Synthesis and characterisation information of CNAzO14; 2. Preparation of 6BP18; 3. Preparation of EV2ON(Tf)_2_; 4. Thermal and liquid crystal properties of CNAzO14; 5. Additional figures: Figure S1. ^1^H-NMR of CNAzO14 [400 MHz, CDCl_3_]; Figure S2. POM mesophase texture of 6BP18 at 100 °C, obtained from a cooling process, and DSC thermograms [first (1) and second (2) heating (H) and cooling (C) cycles obtained at a scanning rate of 10 °C min^−1^]; Figure S3. POM mesophase texture of CNAzO14 at 91 °C, obtained from a cooling process, and DSC thermograms [second heating/cooling cycles at a scanning rate of 10 °C min^−1^]; Figure S4. Photograph showing the experimental setup used during the combined microscopic, dielectric, and light irradiation tests; Figure S5. Bode plots of the permittivity loss factor (e″) obtained in isothermal steps upon cooling from the isotropic melts, corresponding to (a) 5%-CNAzO14/6B18 and (b) 50%-CNAzO14/Ev2ON(Tf)_2_; Figure S6. Polarised optical micrographs (POMs) obtained for 5%-CNAzO14/6B18 (a) before and (b) during UV irradiation at 365 nm (130 °C). White bars correspond to 20 mm.
