# Supplementary material for: Modulating the Conductivity of Light-Responsive Ionic Liquid Crystals
Source: Molecules. 2024 Sep 20;29(18):4459. doi: 10.3390/molecules29184459 (PMC11434579; doi:10.3390/molecules29184459)
Supplement: Supplementary file 1 [file molecules-29-04459-s001.zip › molecules-3174113-supplementary.pdf]

Supporting information

Modulating the conductivity of light-responsive ionic liquid crystals.

Umama Bendaoud[1], Pradip K. Bhowmik[2], Si L. Chen[2], Haesook Han[2], Seonghyeok L. Cox[2], Jasmin Liebsch[1,3], M. Blanca Ros[4], Thamil Selvi Velayutham[5], Nurul Fadhilah Kamalul Aripin[6,7], and Alfonso Martinez-Felipe[1,*]

[1] Chemical Processes and Materials Research Group, School of Engineering, Centre for Energy Transition, University of Aberdeen, King's College, Old Aberdeen AB24 3UE, UK.

[2] Department of Chemistry and Biochemistry, University of Nevada Las Vegas, 4505 S. Maryland Parkway, Box 454003, Las Vegas, NV 89154, USA.

[2] Institute of Biological Sciences, Faculty of Science, University of Malaya, 50603 Kuala, Lumpur, Malaysia.

[3] Department of Chemistry, University of Aberdeen, King's College, Old Aberdeen AB24 3UE, UK

[4] Instituto de Nanociencia y Materiales de Aragón, Departamento de Química Orgánica, Facultad de Ciencias, Universidad de Zaragoza-CSIC, Campus San Francisco, E-50009 Zaragoza, Spain.

[5] Low Dimensional Materials Research Center, Department of Physics, Faculty of Science, Universiti Malaya, 50603 Kuala, Lumpur, Malaysia.

[6] School of Chemical Engineering, College of Engineering, Universiti Teknologi MARA, 40450 Shah Alam, Selangor, Malaysia.

[7] Department of Chemistry, School of Natural and Computing Sciences, University of Aberdeen, King's College, Aberdeen AB24 3UE, Scotland, UK.

*To whom correspondence should be addressed. E-mail: a.martinez-felipe@abdn.ac.uk

Contents

1. Synthesis and chacaterisation information of CNAzO14
2. Preparation of 6BP18
3. Preparation of EV2ON(Tf)$_2$
4. Thermal and Liquid Crystal Properties of CNAzO14
5. Additional figures

1. Synthesis and chacaterisation information of CNAzO14

1.1. Materials and Methods

Chemical reagents were purchased from Aldrich and were used without further purification. Synthetic procedures and characterization data of the novel compounds are reported in section 2. Analytical TLC was performed on 60F 254, 60A-15 µm silica gel polyester plates (Merck). NMR spectra were recorded on spectrometer Brucker AV-400 operating at 300.13 MHz for ^{1}H and 100.62 MHz for ^{13}C. The chemical structure of the synthesized compounds was determined by elemental analysis performed on a Perkin-Elmer 2400 CHNS elemental analyzer. FT-IR spectra were performed in a ThermoNicolet Avatar 360 using KBr pellets. Mass spectral data were obtained using a Brucker MicroTOF-Q. The mesophase identification was based on microscopic examination of the focal-conic and homeotropic textures formed by samples between two glass plates. Nikon and Olympus BH-2 polarizing microscopes equipped with a Linkam THMS600 hot stage were used. The temperatures and enthalpies of the phase transitions were determined by calorimetric measurements with DSC TA Instrument Q-20 system.

1.2. Synthetic scheme and experimental details of the synthesis of CNAzO14.

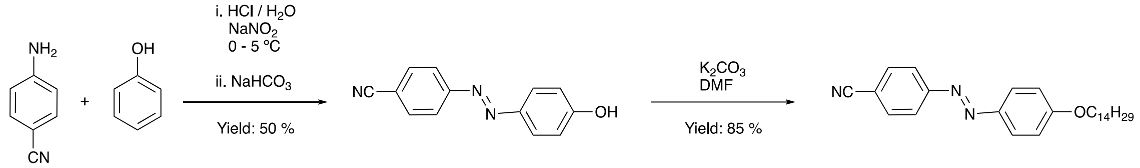

Scheme S1. Synthetic route for the preparation of **CNAzO14**

CNAzO14 was synthesized by the diazocoupling reaction of 4-aminobenzonitrile with phenol followed by the alkylation of the hydroxyl group.

4-Cyano-4'-hydroxiazobenzene: 4-aminobenzonitrile (15 g, 130 mmol) were solved in 80 mL of hydrochloric acid (37%) and 240 mL of water. To the resulting stirred mixture cooled at 0 °C was added, dropwise, a solution of sodium nitrite (10.5 g, 150 mmol) in 100 mL of water. The reaction was stirred for 30 minutes keeping the temperature below 5 °C. The resulting diazonium chloride was coupled with phenol (14.3 g, 150 mmol) with stirring for 1 h, keeping the temperature below 5 °C. $NaHCO_3$ was then added until the solution was neutralized. The azo-compound which formed precipitate was filtered and washed several times with water. The product was purified by recrystallization from cold ethanol, precipitate was filtered and dried in vacuum. Yield 13 g (45%). Dark red solid, m. p.: 206 °C; (203-205°C in Han, Y. K.; Dufour, B.; Wu, W.; Kowalewski, T.; Matyjaszewski, K. *Macromolecules* 2004, 37, 9355-9365)

^{1}H NMR (400 MHz, $CDCl_3$) δ (ppm) = 7.92 (d, J=8.5Hz, 2H, Ar-H), 7.90 (d, J=8.5Hz, 2H, Ar-H), 7.80 (d, J=8.8Hz 2H, Ar-H), 6.96 (d, J=8.8Hz 2H, Ar-H), 5.30 (broad signal, 1H).
^{13}C NMR (400 MHz, $CDCl_3$) δ (ppm) = 144.2, 132.3, 129.7, 128.7, 117.4, 115.7, 111.3.
FTIR (KBr, $\tilde{\nu}$ = cm^{-1}): 3456 (-OH), 2238 (CN), 1604 (C=C, Ar), 1586 (C=C, Ar).

4-Cyano-4´-n-tetradecyloxyazobenzene: To a suspension of 4-cyano-4'-hydroxiazobenzene (1.06 g, 4.8 mmol) in 20 mL of DMF were added dried potassium carbonate (0.98 g, 7.2 mmol). The mixture was stirred for 1 h followed by the addition of 1-bromotetradecane (1.7 g, 6.2 mmol) dropwise with constant stirring. The resulting mixture was stirred under reflux for 8 h, and progress of the reaction was monitored by TLC. it is then cooled to room temperature. Subsequently, 200 mL of water are added and the aqueous phase was extracted three times with a hexane/ethyl acetate mixture (1:1), the organic phase was washed with water, dried with $MgSO_4$, and evaporate to dryness. Purification was carried out by crystallization in ethanol. Yield: 1.7 g, 85%. Yelow solid. Cr 105.6 °C [61.1 kJ mol^{-1}] I / I 105.2 °C [4.6 kJ mol^{-1}] SmA 82.5 °C [54.5 kJ mol^{-1}] Cr

^{1}H NMR (400 MHz, $CDCl_3$) δ (ppm) = 7.94 (d, J=9.3Hz 2H, Ar-H), 7.92 (d, J=9.3Hz, 2H, Ar-H), 7.78 (d, J=8.6Hz, 2H, Ar-H), 7.01 (d, J=9.1Hz, 2H, Ar), 4.05 (t, J=6.6, 2H, $ArOCH_2$), 1.83 (d, 2H, $-CH_2$), 1.24-1.48 (m, 22 H, $-(CH_2)_{11}$), 0.88 (t, 3H, CH_3).

^{13}C NMR (400 MHz, $CDCl_3$) δ (ppm) = 14.1, 22.7, 25.9, 29.3, 68.7, 114.3, 115.7, 117.4, 123. 6, 128.7, 129.7, 132.3, 144.2, 157.0, 161.6

FTIR (KBr, $\tilde{\nu}$ =cm^{-1}): 2925, 2865 (C-H), 2226 (CN), 1606 (C=C, Ar), 1552 (C=C, Ar), 1248(C–O).

MSI+ HRMS: m/z 442,29 [M+Na]+.

Elemental Analysis: Experimental (Calculated) data for $C_{27}H_{37}N_3O$: C, 77.3 (77.5%); H, 8.89 (9.04%); N, 10.01 (9.98%).

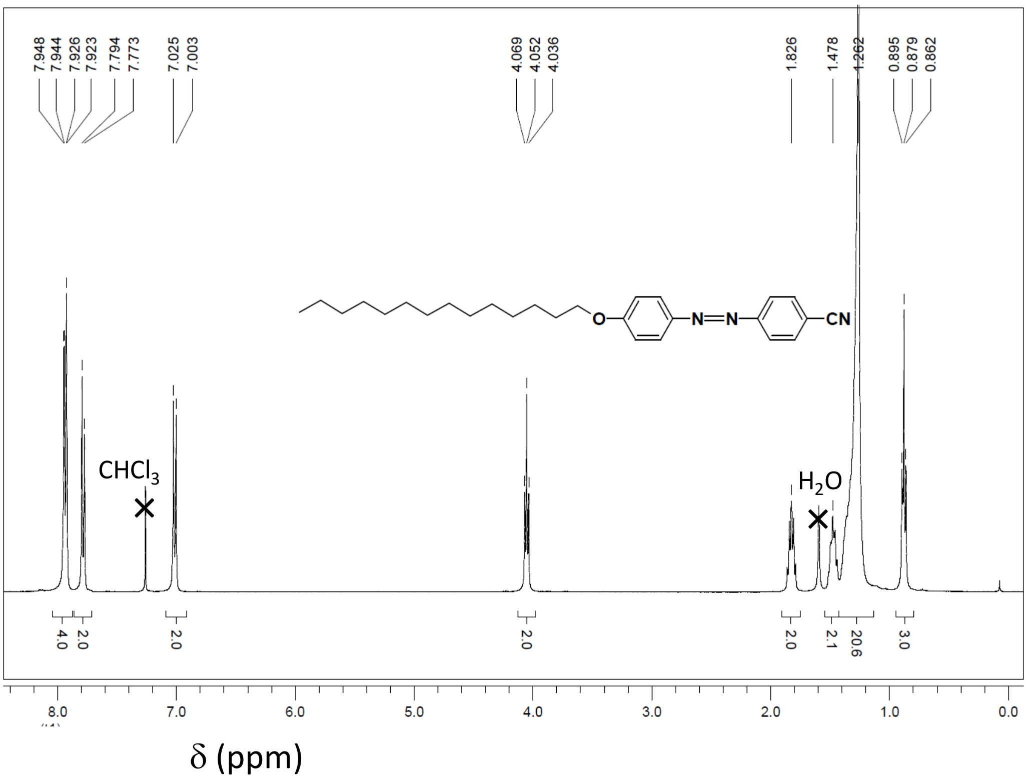

Figure S1.^{1}H-NMR of **CNAzO14** [400 MHz, $CDCl_3$].

2. Preparation of 6BP18

(Bhowmik, P. K.; Noori, O.; Chen, S. L.; Han, H.; Fisch, M. R.; Robb, C. M.; Variyam, A.; Martinez-Felipe, A. *J. Mol. Liq* 2021, *328,* 115370)

2.1 Preparation of 1-hexyl-4,4′-bipyridinum bromide (BP6Br)

The 4, 4′-bipyridine (0.821g, 1.69 mmol) was dissolved in 25 mL of acetonitrile (ACN); 1-bromohexane, (0.335 g, 2.03 mmol) were added dropwise slowly to the reaction flask and the solution was heated in an oil bath at 81 for 24 h . Reaction was monitored using an alumina TLC plate and an eluant of a mixture of acetone and methanol (4/1, v/v). Once the reaction was complete, the reaction was turned off and reaction content was air-filtered to remove yellow dialkylated product. Acetonitrile was removed from the filtrate using a rotary evaporator to yield the crude product which was then washed with toluene. Finally, it was purified by using an alumina column chromatography and an eluent of a mixture acetone and methanol (4/1 v/v) giving a pure light gray/white compound (0.346 g, 1.10 mmol, yield 64%). δ_H (CD_3OD, 400 MHz, ppm): 9.14-9.16 (2H, d, *J* = 6.8 Hz), 8.83-8.85 (2H, dd, *J* = 1.7 Hz, *J* = 4.7 Hz), 8.53-8.55 (2H, d, J = 6.8 Hz), 8.00-8.02 (2H, dd, J = 1.7 Hz, J = 4.7 Hz), 4.69-4.72 (2H, t, *J* = 7.2 Hz), 2.05-2.12 (2H, m), 1.36-1.48 (6H, m) 0.92-0.96 (3H, m).

2.2 Preparation of 1-hexyl-1′-octadecyl-4,4′-bipyridinium dibromide ($6BP18Br_2$)

An amount of BP6Br (0.500 g, 1.56 mmol) and excess of 1-bromooctadecane (1.140 g, 3.42 mmol) were added to 5 mL of ACN and heated to reflux for 120 h. The completion of the reaction was monitored by using an alumina gel thin layer chromatography and an eluent of a mixture of acetone and methanol (4/1 v/v). The reaction mixture was then cooled to room temperature (rt) and the crude product was collected by filtration. It was purified by simply washing with ACN and hexane, respectively, giving the pure product (0.910 g, 1.39 mmol, yield = 89%). δ_H (CD_3OD, 400 MHz, ppm): 9.29-9.31 (4H, d, *J* = 6.8 Hz), 8.70-8.71 (4H, d, *J* = 6.4 Hz), 4.75-4.79 (4H, t, *J* = 7.6 Hz), 2.09-2.13 (4H, m), 1.39-1.47 (36H, m) 0.92-0.99 (6H, m).

2.3. Preparation of 1-hexyl-1-octadecyl-4,4′-bipyridiniumdi[bis(trifluoromethanesulfonyl)imide] (6BP18)

The procedure was described as follows. An amount of $6BP18Br_2$ (0.694 g, 1.06 mmol) was dissolved in 15 mL of methanol on heating; 5 mL of a methanol solution of $LiNTf_2$ (0.699 g, 2.33 mmol) was then added slowly dropwise over a period of two min. The reaction flask was then heated to reflux for 48 h. At the end of the reaction period, methanol was removed using a rotary evaporator. Then 30 mL of water was added and heated to boiling resulting in solid product, which was then filtered to get the crude product. It was then washed repeatedly with water and dried in vacuum oven at 80 °C giving the desired product (1.012 g, 0.96 mmol, yield = 90%). δ_H (CD_3OD, 400 MHz, ppm): 9.13-9.12 (4H, d, *J* = 6.0 Hz), 8.51-8.53 (4H, d, *J* = 6.8 Hz), 4.61-4.64 (4H, t, *J* = 7.6 Hz), 1.95-2.02 (4H, m), 1.26-1.33 (36H, m), 0.78-0.85 (6H, m). δ_C (CD_3OD, 400 MHz, ppm): 149.99, 145.58, 126.88, 124.50, 121.31, 118.12, 114.94, 61.95, 31.64, 31.13, 31.07, 30.84, 29.35, 29.32, 29.29, 29.21, 29.04, 28.70, 25.78, 25.43, 22.30, 22.00, 13.01, 12.82. Anal. calcd for $C_{38}H_{58}N_4O_8F_{12}S_4$ (1055.13): C, 43.26; H, 5.54; N, 5.31; S, 12.16%. Found C, 43.23; H, 5.57; N, 5.25; S, 12.23%.

3. Preparation of $EV2ON(Tf)_2$

(Bhowmik, P. K.; Chen, S. L.; Han, H.; Ishak, K. A; Velayutham, T. S..; Bendaoud, U.; Martinez-Felipe, A. *J. Mol. Liq* 2022, *365,* 120126)

3.1 Synthetic procedure for 4-(2-ethoxyethoxy) aniline

The 4-(2-ethoxyethoxy) aniline was prepared according to the slightly modified literature procedure (Sudhakar, S.; Narasimhaswamy, T.; Srinivasan, K. S. V. *Liq. Cryst.* 2000, *27,* 1525-1532). The modification was the use of acetone instead of ethanol and of 2-ethoxyethyl bromide instead of 2-ethoxyethoxy tosylate in the alkylation of 4-hydroxyacetanilide. The 2-ethoxyethyl bromide was prepared via Appel reaction (Step 1) (Kim, I.-H.; Tsai, H.-J.; Nishi, K.; T. Kasagami, T.; Morisseau, C.; Hammock, B. D. *J. Med. Chem.* 2007, *50,* 5217-5226). The synthesis of 4-(2-ethoxyethoxy) aniline is described as follows. It was prepared in a three-step reaction starting with the bromination of 2-ethoxyethanol via Appel reaction [S3]. The synthesis of 2-ethoxyethyl bromide is as follows. An amount of 2-ethoxyethanol (0.505 g, 5.61 mmol) and an excess of triphenylphosphine (2.458 g, 9.37 mmol) were added to an Erlenmeyer flask and dissolved in 10 mL of dichloromethane (DCM). When the mixture was stirred and cooled to 0 °C, carbon tetrabromide (2.326 g, 7.01 mmol) dissolved in 10 mL of DCM was added to the mixture dropwise. Once all the carbon tetrabromide solution was added, the reaction mixture was let stir at room temperature for 30 min. After 30 min., the DCM was removed using a rotary evaporator. Upon removal of the DCM, 30 mL of hexane was added to the reaction mixture to precipitate out the excess starting material and byproducts. The reaction mixture in hexane was cooled down to -77 °C by keeping the flask in an isopropyl alcohol and dry ice bath. The contents of the flask were filtered through Celite, and the hexane was evaporated leaving a pure product of 2-ethoxyethyl bromide (0.444 g, 2.90 mmol, Yield = 52%).

In the Step 2, the alkylation of 4-hydroxyacetanilide was performed as follows. 2- ethoxyethyl bromide (0.444 g, 2.90 mmol) was added to a round-bottomed flask containing 4- hydroxyacetanilide (0.483 g, 3.19 mmol) dissolved in 50 mL of acetone. Potassium carbonate (0.401 mg, 2.90 mmol) was added to the flask and the reaction mixture was heated to reflux on stirring for 24 h. At the end of the reaction, the mixture was brought to room temperature and filtered. The acetone was removed using a rotary evaporator and the product was purified by extraction with DCM and warm deionized water. The DCM was then evaporated to yield a pure product of 4-(2-ethoxyethyl) acetanilide (0.574 g, 2.57 mmol, Yield = 89%).

Finally, a hydrolysis reaction (Step 2) was performed by adding 4-(2-ethoxyethyl) acetanilide (0.574 g, 2.57 mmol) to a three-necked flask with sodium hydroxide (2.000 g, 50.0 mmol) dissolved in 25 mL of deionized water. The reaction flask was heated under nitrogen atmosphere for 12 h. At the end of the reaction, the flask was cooled down to room temperature and the desired product was purified by extraction with DCM and deionized water. The DCM was removed using a rotary evaporator to yield a pure product of 4-(2-ethoxyethyl) aniline (0.388 g, 2.14 mmol, Yield = 83%).

3.2 Synthetic procedure for Zincke salt

It was prepared, according to the literature, from the reaction of 1- chloro-2,4-dinitrobenzene (2.5 equivalents) with 4,4′-bipyridine (1 equiv.) on heating in acetonitrile (Step 3) (Sharma, G. D.; Saxena, D.; Roy, M. S. *Synth. Met.* 1999, *106,* 97-105).

3.3 Synthetic procedure for bis-(4-ethylethyleneneoxyphenyl)-4,4′-bipyridinium dichloride (P1)

In Step 4 P1 was prepared by adding the 4-(2-ethoxyethyl) aniline (0.154 g, 0.85 mmol) to a round-bottomed flask containing Zincke salt (0.217 g, 0.39 mmol) and 15 mL of N,N-dimethylacetamide (DMAc). The reaction mixture was stirred at room temperature for 3 h. At the end of the reaction, the crude product was collected by simply gravity filtration and washed with acetone to give a pure product (0.139 g, 0.25 mmol, yield = 64%).

3.4 Synthetic procedure for bis-(4-ethoxyethyleneoxyphenyl)-4,4′-bipyridinium bis(triflimide) by metathesis reaction (EV2ON(Tf)$_2$))

The salt was synthesized from the metathesis reaction of P1 with lithium triflimide (Step 5). The lithium salt (0.316 g, 1.10 mmol) dissolved in 5 mL of deionized water was added to a reaction flask containing a clear solution of P1 (0.245 g, 0.44 mmol) dissolved in 20 mL of ethanol. The flask was heated to reflux for 72 h. At the end of the reaction, the solvent ethanol was removed by using a rotary evaporator. The reaction mixture was then dissolved in chloroform and extracted from deionized water to give a pure brown product (0.429 g, 0.41 mmol, yield = 93%). FTIR (NaCl, ν_{max} cm^{-1}): 3117, 3071, 2978, 2932, 2870, 1628, 1597, 1450, 1342, 1180, 1126, 1049, 826, 787, 733, 610, 571, 501. δH (CD3OD, 400 MHz, ppm): 9.46 (4H, br), 8.81 (4H, br), 7.84 (4H, d, *J* = 8.4), 7.33 (4H, d, *J* = 9.2), 4.29 (4H, t, *J* = 4.8), 3.87 (4H, t, *J* = 2.8), 3.65 (4H, t, *J* = 7.2) 1.25 (6H, t, *J* = 7.2). δC (CD3OD, 100 MHz, ppm): 163.20, 146.83, 128.27, 126.91, 122.77, 119.58, 117.44, 69.95, 69.52, 67.86, 15.46. δF (CD3OD, 376 MHz, ppm): -80.14. Anal. Calc for $C_{34}H_{34}F_{12}N_4O_{12}S_4$ (1046.89): C, 39.01; H, 3.27; N, 5.35; S, 12.25%. Found C, 39.17; H, 3.19; N, 5.46; S, 12.25%.

4. Thermal and Liquid Crystal Properties of CNAzO14.

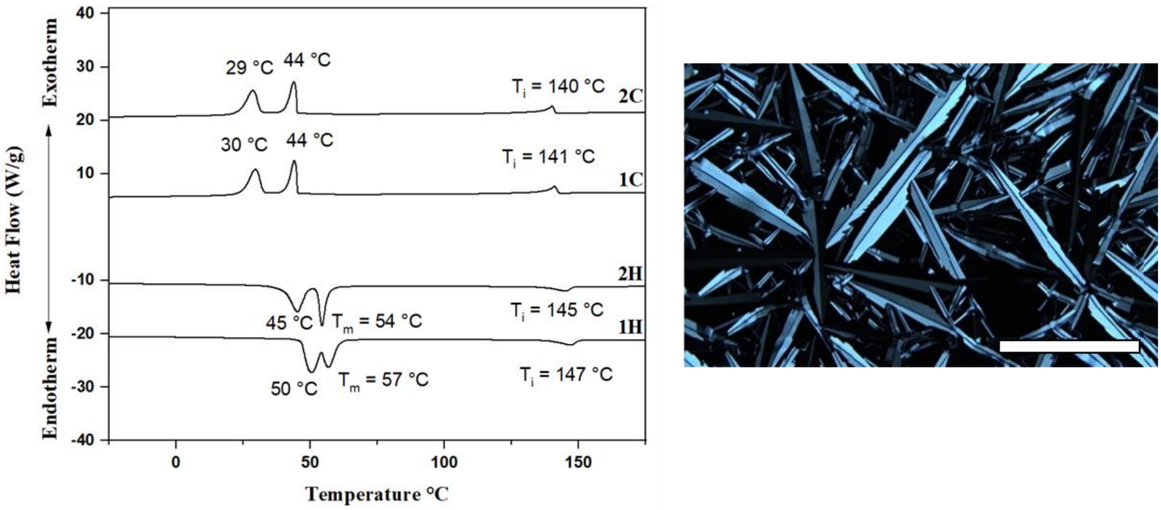

Figure S2. POM mesophase texture of 6BP18 at 100°C, obtained from a cooling process, and DSC thermograms [first (1) and second (2) heating (H) and cooling (C) cycles obtained at a scanning rate of 10 °C min^{-1}]

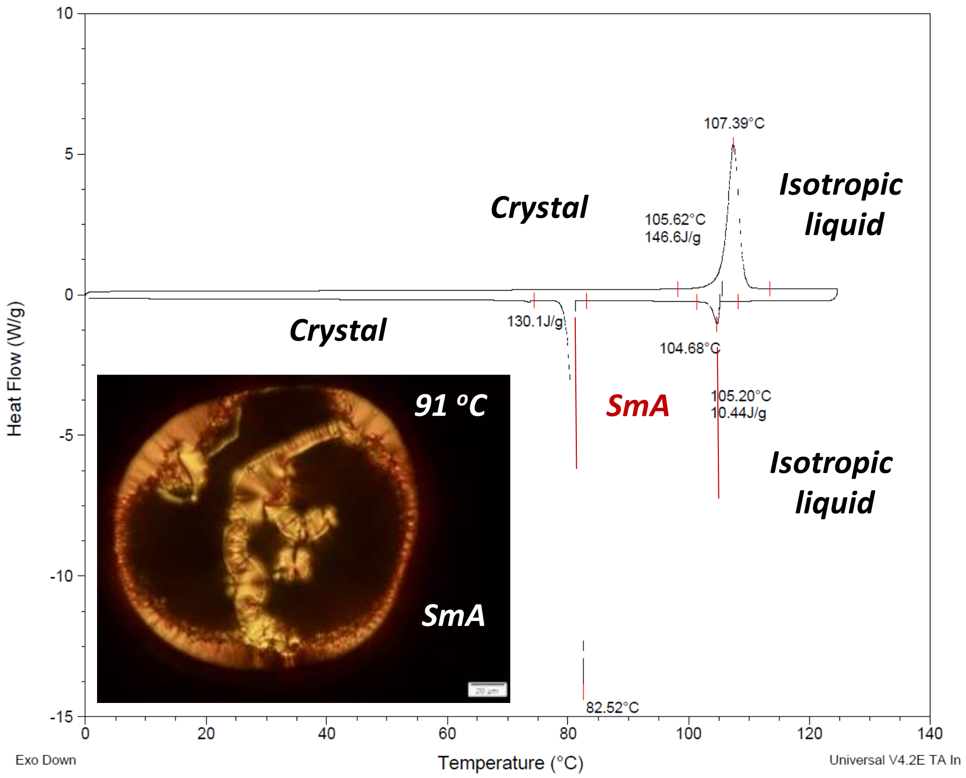

Figure S3. POM mesophase texture of CNAzO14 at 91 °C, obtained from a cooling process, and DSC thermograms [second heating/cooling cycles of at a scanning rate of 10 °C min^{-1}]

.

5. Additional figures

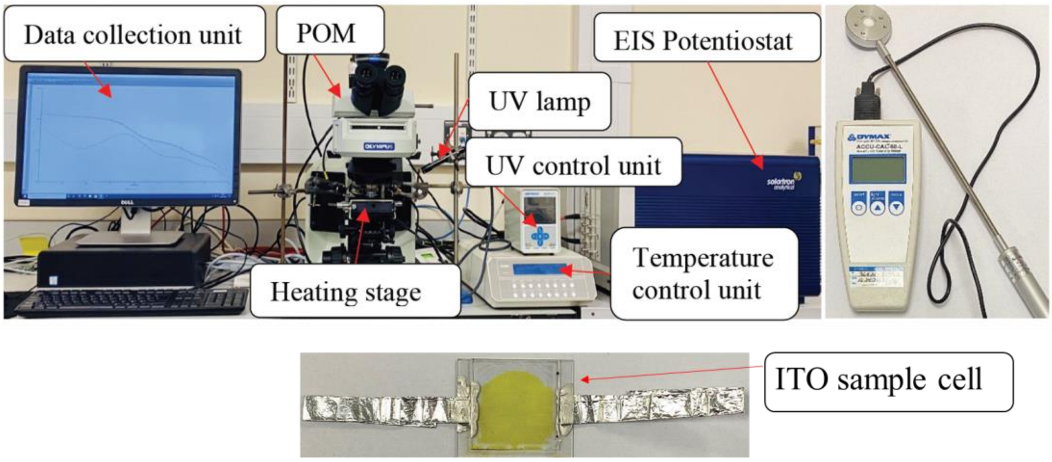

Figure S4. Photograph showing the experimental setup used during the combined microscopic, dielectric, and light-irradiation tests.

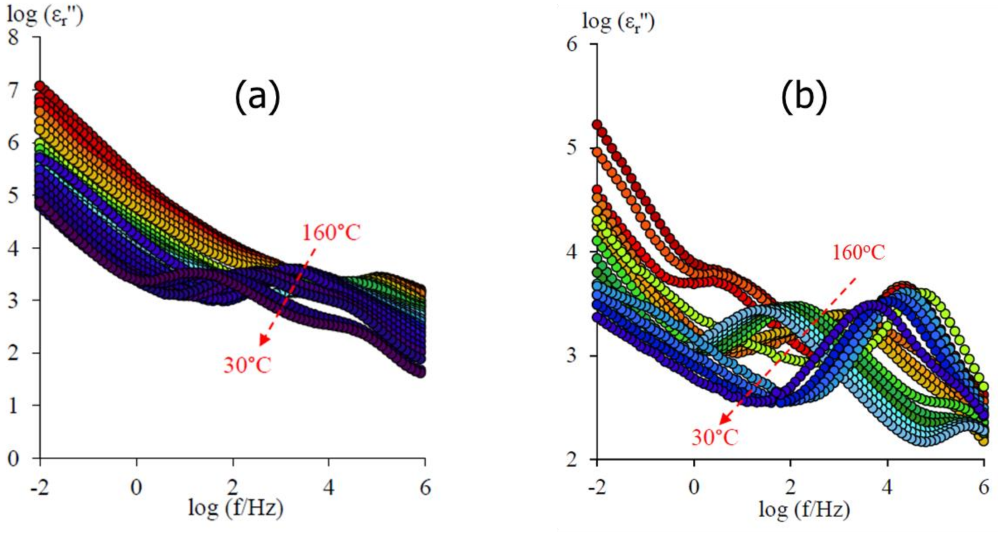

Figure S5.. Bode plots of the permittivity loss factor (ε'') obtained in isothermal steps on cooling from the isotropic melts, corresponding to: **(a)** 5%-CNAzO14/6B18 and **(b)** 50%-CNAzO14/Ev2ON(Tf)$_2$.

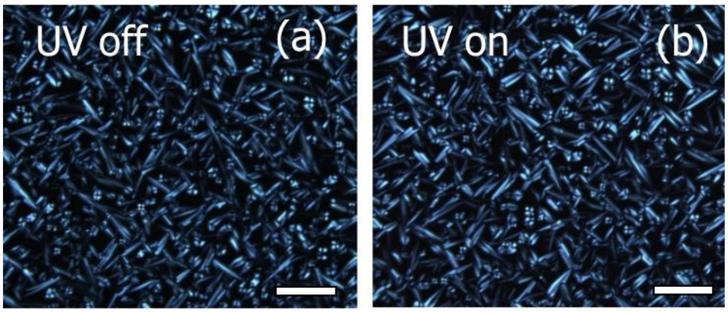

Figure S6. Polarised optical micrographs (POM) obtained for 5%-CNAzO14/6B18: **(a)** before; and **(b)** during UV irradiation at 365 nm (130°C). White bars correspond to 20 μm.
